# Social Message Account or Processing Conflict Account – Which Processes Trigger Approach/Avoidance Reaction to Emotional Expressions of In- and Out-Group Members?

**DOI:** 10.3389/fpsyg.2022.885668

**Published:** 2022-07-28

**Authors:** Dirk Wentura, Andrea Paulus

**Affiliations:** Department of Psychology, Saarland University, Saarbrücken, Germany

**Keywords:** emotional expression, approach/avoidance, behavioral reaction, social message, processing conflict

## Abstract

Faces are characterized by the simultaneous presence of several evaluation-relevant features, for example, emotional expression and (prejudiced) ethnicity. The social message account (SMA) hypothesizes the immediate integration of emotion and ethnicity. According to SMA, happy in-group faces should be interpreted as benevolent, whereas happy out-group faces should be interpreted as potentially malevolent. By contrast, fearful in-group faces should be interpreted as signaling an unsafe environment, whereas fearful out-group faces should be interpreted as signaling inferiority. In contrast, the processing conflict account (PCA) assumes that each face conveys two rather independent evaluative features, emotion and ethnicity. Thus, stimuli might be either affectively congruent or incongruent, and thereby exert influences on behavior. The article reviews the evidence with regard to the two accounts before reporting an experiment that aims at disentangling them. In an approach/avoidance task (AAT), either happy/fearful faces of German and Turks were presented or happy/fearful faces of young and old persons. There are prejudices against Turk/Middle-eastern persons (in Germany) as well as against old persons. For SMA, the two prejudices are of different type; thus prediction for the AAT diverge for the two group conditions. In contrast, for PCA both group features (i.e., Turk ethnicity and old age) are negative features (in comparison to their counterparts) which are affectively congruent or incongruent to the emotional expression. Hence, the results pattern in the AAT should be comparable for the two group conditions. In accordance with SMA but in contrast to PCA, we found different patterns for the two group conditions.

## Introduction

Faces are characterized by the simultaneous presence of several evaluation-relevant features, for example, emotional expression, ethnicity, or age. The (seemingly) simple question to be addressed in this article is whether these features – particularly emotional expression and ethnicity – are initially processed independently (and may interact at later stages of processing to influence behavior) or whether they are immediately integrated to form a social message based on both pieces of information.

There are affirmative answers for both positions. The social message account (SMA; Weisbuch and Ambady, 2008) hypothesizes the immediate integration of emotion and group membership to form social messages. For example, while the smile of a member of one’s own group may be interpreted by default as a signal of belonging, the smile of a member of an ethnic group that is met with prejudice (e.g., Blacks in the White majority) may be immediately interpreted as condescension. The processing conflict account (PCA; Kozlik and Fischer, 2020) assumes independent extraction of the two valent features, that is, a smile is a positive feature whereas ethnicity (of a group that is met with prejudice) is a negative feature. At a later stage, the features then potentially interact due to their affective congruence or incongruence. We will elaborate on both accounts.

The importance of distinguishing between the two accounts is obvious: To address only one point, both accounts claim that the initial involuntary reaction to a smiling outgroup member will be negative (which entails the risk of stabilizing the prejudice). However, according to PCA the smile is still a (positively evaluated) smile. Thus, PCA involves the possibility that this feature will eventually dominate the communication situation and thereby help to overcome the prejudice. According to SMA, the smile itself is negatively connoted which might lead to a sobering vicious circle of maintaining the prejudice.

### The Social Message Account

According to SMA, emotional expressions communicate a social message to the perceiver of the emotion. Happy expressions, for example, typically signal a safe environment and/or a desire to affiliate whereas fearful faces can be interpreted as signaling an unsafe social environment. This social message, however, is influenced by the social situation: Depending on features like the relationship between expresser and perceiver of the emotion, the same emotional expression might be seen as sending a different message: if the happy face is, for example shown by a member of a potentially dangerous out-group, it might be interpreted as potentially malevolent (i.e., signaling rival superiority). A fearful out-group face, by contrast, might be interpreted as signaling inferiority. Thus, SMA emphasizes the social function and interpersonal consequences of facial expressions (Weisbuch and Ambady, 2008).

There are several studies employing different paradigms that provided evidence for the hypothesized ethnicity × emotion interaction. Since it is most relevant for the present study, we start with the approach–avoidance paradigm: Paulus and Wentura (2014) used this approach for finding evidence for SMA. In their experiments, participants had to push the mouse device of the computer away from them or pull it toward them depending on whether the stimulus presented on the screen was slightly blurred on the left or right side. Emotional expressions (i.e., happy and fearful faces) shown by in-group and out-group members were employed as stimuli. Group membership was implemented by showing images of White–Caucasian and Turkish/Middle-Eastern young men. The results supported the SMA: for in-group faces (i.e., White–Caucasian for participants of the German majority), the default effect of this paradigm appeared: happy faces were relatively faster approached than avoided, compared to fearful faces. However, the pattern reversed for out-group faces (i.e., Turkish/Middle-eastern young men). The effect was conceptually replicated in a second study with a modified minimal group manipulation.

As already mentioned, the SMA was also supported by other paradigms: Recently, Gurbuz et al. (2022) used the Extrinsic Affective Simon Task (EAST; De Houwer, 2003). In the EAST, participants had two tasks: If the stimulus was a word (always a clearly positive or negative one), participants had to categorize its valence by pressing either a positive or negative key. If the stimulus was a face, participants had to categorize whether the left or right side of the face was blurred. Importantly, the same two keys were used for both tasks. The basic idea of the EAST is that keys acquire the evaluative meaning during the word trials. As a consequence, participants categorize an arbitrary feature of the faces (i.e., the side of blurredness) by evaluatively loaden responses (comparable to saying aloud “Good!” and “Bad!”). The affective Simon effect (De Houwer and Eelen, 1998) consists in faster responses if the task-irrelevant valence of a stimulus matches the evaluatively loaden response compared to the non-matching condition. In their experiment, Gurbuz et al. (2022) presented emotional expressions shown by in-group and out-group members. They found the expected emotion × ethnicity × valence interaction, which was predicted by the SMA: a larger positivity score (i.e., the difference in response times for negative responses minus response times for positive responses) for happy faces compared to fearful faces was found of for White Caucasian stimulus persons; this difference was numerically reversed for Turkish/Middle-Eastern faces.

Furthermore, Paulus et al. (2019) provided evidence for the SMA in the startle paradigm. In the startle paradigm, the human eyeblink reflex is (typically) elicited by the presentation of a loud stimulus; the size of the eyeblink reflex is then examined as the dependent variable. Interestingly, this magnitude is influenced by the emotional state of the “blinking” person: In a positive, relaxed emotional state, the eyeblink reflex is diminished. However, in a negative emotional state, the reflex is enhanced. In two experiments, Paulus et al. (2019) examined the modulation of the startle response by happy, fearful, and angry expressions shown by in-group and out-group members. As predicted from SMA, an interaction between group membership and emotional expression emerged, such that happiness expressed by an in-group member resulted in lower startle responses compared to the same expression shown by an out-group member; the opposite pattern emerged for fearful and angry expressions.

However, there also exist mixed results regarding the SMA: Weisbuch and Ambady (2008) used the evaluative priming paradigm (Fazio et al., 1986) with happy and fearful faces of different ethnicities (i.e., White–Caucasian vs. African–American) as prime stimuli. In this paradigm, primes precede positive or negative target stimuli that have to be categorized accordingly. If primes are clearly positive and negative as well, a congruence effect will be typically found: positive/positive as well as negative/negative pairs will yield faster responses than the reversed pairings (see Herring et al., 2013, for a meta-analysis). If primes of unknown or assumed valence are presented, one can – with some caution – infer valence of primes from priming effects. Weisbuch and Ambady found priming effects for in-group primes that conform to the nominal evaluation of emotions (i.e., happy/fearful faces acted as positive/negative stimuli, respectively). However, the effect reversed for out-group faces (i.e., in this case happy/fearful faces acted as *negative/positive* stimuli, respectively). Thus, the automatically processed valence of emotional expressions reflects the expectations of SMA.

However, in two independent studies, the evaluative priming results found by Weisbuch and Ambady (2008) could not be replicated: Craig et al. (2014) found no interactive influence of emotion and group in the evaluative priming paradigm (i.e., no evidence for the signature of the SMA) but a main congruence effect of emotion with regard to priming (i.e., happy/fearful primes acted as positive/negative primes, irrespective of group). Paulus and Wentura (2018) confirmed the absence of an interactive influence of emotion and group; they found emotion congruence effects (as Craig et al., 2014) as well as group congruence effects (i.e., in-group/out-group primes acted as positive/negative primes, independent of emotion).

In summary, there is strong evidence for the assumptions of the SMA. However, as the review above clearly shows, also some results did not confirm the SMA. Moreover, recently Kozlik and Fischer (2020) claimed that the results pattern typically explained by SMA (e.g., the approach/avoidance pattern found by Paulus and Wentura, 2014) can be reinterpreted by an alternative account.

### The Processing Conflict Account

Basically, Kozlik and Fischer (2020) argue that, typically, each face conveys two rather independent evaluative features: First, the emotional expression that is either positive (e.g., a smile) or negative (e.g., a fearful or angry expression); second, the identity feature of the face that is either positive (e.g., an in-group member) or negative (e.g., an out-group member) as well. Hence, stimuli might be either affectively congruent or incongruent, depending on the composition of expression and identity. Depending on the exact task, this congruency status might then influence the dependent variable such that affectively congruent stimuli elicit positive reactions whereas affective incongruent elicit negative reactions. With this account, the approach-avoidance results by Paulus and Wentura (2014) can be reinterpreted: Happy in-group and fearful out-group faces elicit an approach reaction because they are affectively congruent; fearful in-group and happy out-group faces, on the other hand, are affectively incongruent and therefore elicit an avoidance reaction. Moreover, Kozlik and Fischer (2010) added evidence to their account with data from two new paradigms.

To provide independent evidence for their account, Kozlik and Fischer (2020) suggested a paradigm that is structurally equivalent to the Stroop-Paradigm (Stroop, 1935; for a review, see MacLeod, 1991) and other response–conflict paradigms. In a nutshell, in this paradigm participants had to categorize faces with regard to emotion expression as positive or negative. Group membership can be considered a second positively or negatively connoted feature. Thus, although group membership is a task-irrelevant feature it nevertheless might trigger the correct (in case of congruency) or wrong (in case of incongruency) response. Therefore, in incongruent trials, response conflict has to be overcome; hence, responses are slower in incongruent trials compared to the congruent condition. Indeed, Kozlik and Fischer (2020) found this effect in Experiments 1 and 3 with happy and fearful/angry expressions shown by in-group and out-group members as stimuli. Moreover, in Experiment 1 they found evidence for a congruence sequence effect (i.e., a stronger congruence effect for trials that follow congruent trials) a signature of response conflict paradigms (see, e.g., Duthoo et al., 2014). In Experiment 3, they found the congruence effect to be dependent on the proportion of congruent pairs; again, this is a signature of response conflict paradigms (see, e.g., Logan, 1980).

*Prima facie*, SMA would not predict this congruency effect. At second glance, even these results can be discussed from the backdrop of SMA. For two reasons, we postpone this discussion to the discussion section. First, it is a rather complex argument which would be to distractive here. Second and more importantly, the Stroop-like paradigm does not address the kernel of PCA (i.e., that two features of a face are in conflict) because to explain the effects it is sufficient to assume that the group feature is either congruent or incongruent *to the response* needed for a given target (i.e., the negative outgroup feature is not *per se* in conflict with a happy expression; it is the negative response tendency triggered by the group feature that is in conflict with the positive response that has to be intentionally given to categorize the emotion).

This kernel of PCA is better addressed in Experiment 2. The goal was to show that the congruence/incongruence of the two valenced features of the face – namely emotional expression and ethnicity – influence reactions even if valence is not task relevant. Therefore, the authors presented the faces with slight blur on left or right side and participants had to categorize the side of the blurring. Kozik and Fischer (2020) argue that in this task affective incongruence will nevertheless prolong response time; indeed, they found such an effect.

However, we think the effect found by Kozlik and Fischer (2020) in their Experiment 2 can easily be explained by the SMA as well: SMA considers out-group joy and in-group fear as negative stimuli (because of the communicated social message). It has been shown before that negative stimuli might produce unspecific interference with an ongoing task: In the Emotional Stroop task, the print color of words have to be named. There is evidence that negatively valenced words produce longer naming latencies (e.g., Pratto and John, 1991; Williams et al., 1996; Wentura et al., 2000; Frings et al., 2010). Even closer to our context, there are also emotional Stroop studies with colored faces as stimuli (Van Honk et al., 2001; Putman et al., 2004). Van Honk et al. (2001) found slower color-naming latencies for anger compared to neutral faces. (However, the effect was only found for participants high in trait anger; the overall effect was 3 ms and presumably not significant). Putman et al. (2004) found an overall effect of slowed color-naming latencies for anger compared to neutral faces. (It was, however, restricted to masked faces.) Thus, there is some evidence for an emotional Stroop effect for faces with negative expressions. However, effects seem small and fragile. Nevertheless, Kozlik and Fischer (2020) found the effect; it is, however, compatible with PCA as well as SMA.

In their Experiment 4a^1^, Kozlik and Fischer (2020) conducted a conceptual replication of their Experiment 2 by presenting semiprofile faces; now, direction of faces (i.e., right-looking versus left-looking) was the task-relevant feature. The authors argue (and we agree) that SMA should not predict an interaction of emotion and ethnicity because the hypothesized social message is constrained to directed faces. On the contrary, for PCA direction of faces should not make a difference. Table 4 of Kozlik and Fischer (2020) reveals that the congruence effect in Experiment 4a is *M* = –1 ms. Thus, this null result is more compatible with SMA than PCA.

In sum, we can state that there are definitely contexts that do not reveal the processing of social messages: This holds especially for the evaluative priming results, if we consider the more recent results (i.e., Craig et al., 2014; Paulus and Wentura, 2018) as more robust than the initial findings by Weisbuch and Ambady (2008). Other results are compatible with SMA or both accounts. The latter holds especially for the approach/avoidance task (AAT; Paulus and Wentura, 2014). In the following we will present a study that aims at disentangling SMA and PCA with regard to this task. In a nutshell, we enrich the discussion by varying the stimulus materials. The social message assumptions (as outlined above) plausibly hold for out-groups that are victims of a specific type of prejudice (e.g., Turkish/Middle-Eastern young men in Germany are seen as hostile and threatening); other groups are victims of a different type of prejudice (e.g., old people are seen as weak and worthless) which do not cause the same social messages as the first type of prejudice. Thus, SMA will predict a difference in results for the two types of outgroups. In contrast, for PCA both group features (i.e., Turkish/Middle-Eastern ethnicity and old age) are negative features which are affectively congruent or incongruent to the emotional expression. Hence, PCA does not predict a difference.

### The Present Study

The present study has two aims: first, we aim to conceptually replicate the results by Paulus and Wentura (2014) since both theories – SMA and PCA – build on these findings. In the experiments by Paulus and Wentura (2014), participants were instructed to categorize the emotional expressions of White–Caucasian and Turkish/Middle-eastern young men by pushing the computer mouse away (avoidance movement) or pulling it toward themselves (approach movement). In the present study, we used the manikin task (introduced by De Houwer et al., 2001; see also Krieglmeyer and Deutsch, 2010; Paulus and Wentura, 2016), a different paradigm to measure approach and avoidance reactions.

Second, the present study aims at disambiguating the results found with the approach-avoidance paradigm with regard to the two theoretical accounts. To do so, we added the factor *type of group contrast* to the design. In addition to the replication condition (i.e., emotional expressions of White–Caucasian versus Middle-Eastern young men), emotional expressions of young and old persons were presented.

There are established prejudices toward Middle-Eastern young men (in Germany) and old people (“ageism”). Both prejudices were shown not only in explicit measures but in indirect measures as well (Neumann and Seibt, 2001; Degner et al., 2007; Degner and Wentura, 2011; de Paula Couto and Wentura, 2012; Meissner and Rothermund, 2013; de Paula Couto and Wentura, 2017). However, the two prejudices are different with regard to the basal distinction between other-profitability (other-relevance) and self-profitability (possessor-relevance; Peeters, 1983; Peeters and Czapinski, 1990). This distinction is concerned with the fact that the adaptive value of an attribute (and thus its valence) originates from one of two perspectives, being (a) the perspective of the self and (b) the perspective of the other. For example, the negative connotation of the attribute *mean* stems from an unconditional negative value for the interaction partner of a mean person whereas the negative connotation of the attribute *lonely* stems from its unconditional negative value for the lonely individuals themselves (see Degner and Wentura, 2011, for a more extensive discussion).

Specifically, negative group prejudice can be systematized according to the type of negativity that is associated with different groups. For example, when Middle-Eastern or Turkish immigrants in Germany are evaluated negatively, they are associated with other-relevant negativity, i.e., they are characterized as hostile and threatening (e.g., Wagner et al., 2003). On the contrary, when the elderly are negatively evaluated, they are associated with possessor-relevant negativity, supposed they are characterized as worthless, weak, or incompetent (“ageism”; see, e.g., Kite and Johnson, 1988).

Thus, from the perspective of the SMA it is plausible to assume that the critical group × emotion interaction effect will not be found in every intergroup context: The smile of an elderly woman, for example, might not be evaluated as expressing dominance or arrogance. Drawing on Degner and Wentura (2011; see also Degner et al., 2007; Wentura and Degner, 2010a; de Paula Couto and Wentura, 2012), we assumed that the influence of group membership on reactions to emotional expressions might be limited to groups which are associated with other-relevant negativity (see above). The rationale underlying the experiments by Paulus and Wentura (2014) implicitly assumed an other-relevant negativity toward the out-group: We hypothesized that happiness expressed by out-group members elicits avoidance because it is evaluated as signaling dominance of a potentially dangerous, aggressive, dastardly person. The same rationale applies to fearful faces: Shown by a negatively evaluated out-group member, this expression should elicit an approach reaction and positive evaluation because it is seen as an indication of submission. However, if the out-group negativity is of the possessor-relevant type (i.e., groups which are evaluated negatively because of traits that are relevant for the persons themselves), different evaluations of the social message signaled by an expression should occur. Therefore, we compared the influence of an out-group associated with other-relevant negativity (Middle Eastern young men) to the influence of an out-group associated with possessor-relevant negativity (elderly persons).^2^

## Materials and Methods

### Participants

Sixty-two psychology students (48 females and 13 males; 1 missing value) from Saarland University participated in the experiment. The age range was 18–32 years with a median of 20 years. All participants were native White–Caucasians. Participation was compensated by course credits. The data of one further participant were excluded because they erroneously used the number keys on top of the letter keys instead of the number keys on the number pad for responding (see Section “Procedure”).

With regard to power planning and inferential statistics, we followed the following rationale: Since we used a design with several binary repeated-measures factors, any *F*-test in the corresponding ANOVA is associated with *df_*N*_* = 1 and is therefore *equivalent* to a one-sample t-test with *t* = squareroot(*F*), testing the mean of a specific difference variable on deviation from zero (see, e.g., Maxwell et al., 2017). For example, we can start by computing approach scores by subtracting RTs of approach movements from RTs of avoidance movements for each stimulus category. These approach scores indicate the relation between approach and avoidance movements: A higher (lower) approach score indicates relatively more (less) activation of approach compared to avoidance-related behavior. Paulus and Wentura (2014) reported a 2 (in-group vs. out-group) × 2 (happy vs. fearful) interaction with approach scores as dependent variable. From these approach scores we can calculate the critical difference variable of the interaction test: approach scores for happy/in-group and fearful/out-group (averaged) minus approach scores fearful/in-group, happy/out-group (averaged). The one-sample *t*-test testing the mean of this difference variable on deviation from zero is equivalent to the *F*-test of the just mentioned interaction and therefore, of course, equivalent to the 2 (in-group vs. out-group) × 2 (happy vs. fearful) × 2 (approach vs. avoidance) triple interaction with RTs as the dependent variable. This rationale has two advantages over the simple application of an ANOVA: first, given our specific prediction – that is, the mean of the critical difference should be positive – one-tailed testing is appropriate. Second, the difference variables can be easily checked for outliers and deviations from normality.

With *N* = 62, we were able to find an effect of *d*_*Z*_ = 0.32 with power 1–*β* = 0.8 (α = 0.05, one-tailed). This corresponds roughly to the effect size (*d*_*Z*_ = 0.30) of the decisive interaction effect in Experiment 1 of Paulus and Wentura (2014). Note, effects in Paulus and Wentura (2016) – where we already used the manikin task that will also be used here – were on average somewhat larger than the one in Paulus and Wentura (2014). Thus, our study has enough power to replicate the finding by Paulus and Wentura (2014) in the other-relevant negativity block of our study.

According to PCA, for the possessor-relevant negativity block the same effect as for the other-relevant negativity block should be found. One might argue that the power to obtain significant results for both blocks is only 1–*β* = 0.8 × 0.8 = 0.64 if the block-wise effects are as small as *d*_*Z*_ = 0.32. However, from the PCA perspective we can focus on the possessor-relevant negativity block. If we find the effect here, the SMA logic has failed and the PCA logic is corroborated. Hence, the power of 1–*β* = 0.8 still holds.

According to SMA, we expect a null finding for the possessor-relevant negativity block. Of course, in the ideal case, we should find a significant four-way interaction – that is, the critical difference for other-relevant negativity should be significantly larger than the critical difference for possessor-relevant negativity. Power planning for this interaction is inherently vague because we need an estimate of the standard deviation of the critical difference for the possessor-relevant negativity block and an estimate of the correlation of the two critical difference variables (i.e., the one for other-relevant negativity and the one for possessor-relevant negativity). With two plausible assumptions^3^, however, we can relate the *d*_*Z*_ for the other-relevant negativity block to the *d*_*Z*_ of the higher-order interaction by the term square-root(2). That is, if we assume that the higher-order interaction is associated with *d*_*Z*_ = 0.32 (i.e., the effect that we can detect with power 1–*β* = 0.8), we must assume *d*_*Z*_ = 0.32 square-root(2) = 0.45 for the other-relevant negativity block. This is still an effect size below what is typically called “medium-sized” (i.e., *d*_*Z*_ = 0.5) and within the range of expectation given the use of the manikin task (see above). Admittedly, proceeding from *d*_*Z*_ = 0.30 for the other-relevant negativity block (i.e., the result found by Paulus and Wentura, 2014; see above), we can expect – given the two assumptions – only an effect of *d*_*Z*_ = 0.30/square-root(2) = 0.21 for the higher-order interaction. To find an effect of *d*_*Z*_ = 0.21 with power 1-*β* = 0.8 (α = 0.05, one-tailed), *N* = 142 are needed. We decided against this costly solution because for several conceivable constellations (e.g., PCA is correct; the effect sizes found with the manikin task are larger than the one reported in Paulus and Wentura, 2014) the planned sample size provides enough power. Potentially, an outcome which remains too ambiguous must be the starting point for a second study.

### Design

We employed a 2 (negativity of out-group: other vs. possessor-relevant) × 2 (in-group vs. out-group) × 2 (happy vs. fearful) × 2 (approach vs. avoidance) within-participants design. The factor negativity of out-group was varied block-wise (with counterbalanced order). The assignment of in- and out-group to approach and avoidance responses were varied block-wise (with counterbalanced order, nested within negativity of out-group) as well.

### Materials

Pictures of Turkish/Middle-eastern and German/Dutch young men were taken from the Radboud Faces Database (Langner et al., 2010), the Amsterdam Dynamic Facial Expression Set (van der Schalk et al., 2011) and our own collection (Paulus et al., 2011). Pictures of young and old (German) stimulus persons were taken from the FACES Lifespan Database of Facial Expressions (Ebner et al., 2010). A pre-selection of 13 Turkish/Middle-eastern young men, 13 German/Dutch young men, 16 younger persons (8 female), and 16 old persons (8 female) was evaluated in a validation study (*N* = 18 students; 13 women, 5 men; median age = 26 years, range of 20–31 years). Participants were presented with the images of the 58 stimulus persons, separately in two sets, one with the 26 stimuli for the German/Dutch versus Turkish/Middle-eastern experiment and one with the 32 stimuli for the ageism experiment. Presentation order of the two sets was counterbalanced. Each rating block consisted of five parts, with stimulus persons presented once with the joyful and once with the fearful facial expressions in the first two parts and with a neutral facial expression in the third through fifth parts. Within each part, images were presented in randomized order. In the first part, each picture was to be judged with regard to four features of the emotional expression: (a) the primary emotion shown, (b) intensity, (c) unambiguousness, and (d) naturalness. In the second part, the joyful and fearful facial expressions were rated in terms of the dominance and sociability of the stimulus persons. In the third to fifth part, the (neutral-looking) stimulus persons were rated according to their age, their ethnic typicality, and their attractiveness. All ratings (except two, see below) were on a seven-point bipolar scale: intensity: 1-‘very weak’ to 7-‘very intense’; unambigueness: 1-‘very ambiguous’ to 7-‘very unambiguous’; naturalness: 1-‘very posed’ to 7-‘very natural’; dominance: 1-‘very submissive’ to 7-‘very dominant’; sociability: 1-‘very shy’ to ‘very sociable’; typicality: 1-‘typically German’ to 7 ‘typically Middle-eastern’. The primary emotion was a categorization with response categories: joy, disgust, fear, sadness, surprise, contempt, anger, neutral, other. The estimated age of the stimulus person was typed in by participants. On the base of these ratings, we selected 10 stimulus persons for each of the four groups. Appendix Table A1 shows the means (and standard deviations) of the stimulus sets for all ratings.

### Procedure

**FIGURE 1:**
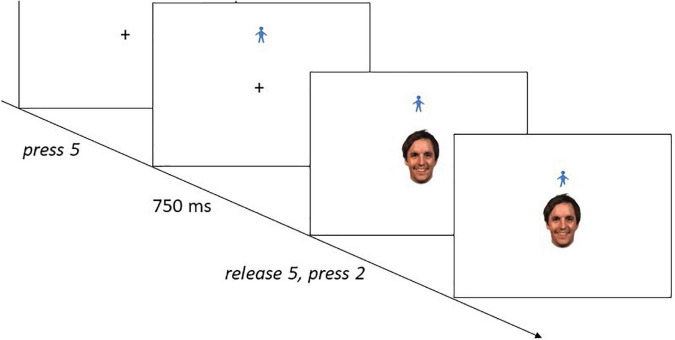
An example of a trial sequence: The participant categorized the German face by moving the manikin toward the face (only the first approach step is shown, see text for further explanations). Facial images sourced from: Paulus et al. (2011). Reproduced with permission.

Up to five individuals were tested in parallel. Participants were seated individually in front of personal computers, separated by partition walls. The procedure (see Figure 1) closely followed the one described in Krieglmeyer et al. (2010), see also Paulus and Wentura (2016): Each trial started with a fixation cross presented in the middle of the screen. Participants were instructed to press and hold the “5” key on the number pad as soon as the fixation cross appeared. Triggered by the key press, a virtual stick figure appeared either below or above the fixation cross. After 750 ms, the fixation cross was replaced by a face stimulus (positioning of the figure was counterbalanced such that the figure appeared above and below each expression equally often). Participants were instructed to move the figure toward or away from the expression by pressing either the “2” or the “8” key on the number pad three times. Each press of “2” moved the figure down 20 pixels and each press of “8” moved it up 20 pixels. Key presses temporarily shortened one of the figure’s legs in an alternate fashion, creating the impression that it walked toward or away from the emotional expression. After three key-presses, the screen turned blank. Participants were instructed to react as quickly and accurately as possible. They received an error message if their reaction was incorrect. The time lapse between the release of “5” and the first press of “2” or “8” was defined as the reaction time (RT).

For the ethnicity part of the experiment, half of the participants first categorized the ethnicity by moving the figure toward German/Dutch faces and away from Turkish/Middle-eastern faces. For the other half of participants, the instructions were reversed. In the second block, the assignment of movement direction to ethnicity was reversed. For the age part of the experiment, half of the participants first categorized the age by moving the figure toward young faces and away from old faces. For the other half of participants, the instructions were reversed. In the second block, the assignment of movement direction to ethnicity was reversed. The ethnicity part and age part of the experiment were assigned in counterbalanced order. Between the two parts a filler task (Zahlen–Verbindungs-Test; Oswald and Roth, 1987) of app. 5 min was administered. Each of the four blocks (ethnicity/age × response assignment) comprised 24 practice trials with neutral-looking faces, four practice trials with happy and fearful faces and 80 main trials.

Each trial started with a fixation cross presented in the middle of the screen. Participants were instructed to press and hold the “5” key on the number pad as soon as the fixation cross appeared. Triggered by the key press, a virtual stick figure appeared either below or above the fixation cross. After 750 ms, the fixation cross was replaced by an emotional expression (positioning of the figure was counterbalanced such that the figure appeared above and below each expression equally often). Participants were instructed to move the figure toward or away from the expression by pressing either the “2” or the “8” key on the number pad three times. Each press of “2” moved the figure up 20 pixels and each press of “8” moved it down 20 pixels. Key presses temporarily shortened one of the figure’s legs in an alternate fashion, creating the impression that it walked toward or away from the emotional expression. After three key-presses, the screen turned blank. Participants were instructed to react as quickly and accurately as possible. They received an error message if their reaction was incorrect. The time lapse between the release of “5” and the first press of “2” or “8” was defined as the RT.

## Results

Trials with an incorrect response (5.6% for German/Turk, 2.8% for young/old) and trials with outlying RTs (i.e., RTs three interquartile ranges above the third or below the first quartile; Tukey, 1977) with respect to the individual distribution (1.5% for German/Turk, 1.7% for young/old) were discarded from the analyses. Mean reaction times are displayed in Table 1. We computed approach scores to enhance comprehensibility of the results. We subtracted RTs of approach movements from RTs of avoidance movements for each stimulus category for each block. These approach scores indicate the relation between approach and avoidance movements: A higher (lower) approach score indicates relatively more (less) activation of approach compared to avoidance-related behavior. Please note that approach reactions were generally faster than avoidance reactions (i.e., a main effect of movement). This effect has repeatedly been reported in the literature (e.g., Rinck and Becker, 2007; Wilkowski and Meier, 2010; Krieglmeyer and Deutsch, 2013; Paulus and Wentura, 2016).

**TABLE 1:** Mean reaction time (RTs in ms; SDs in parentheses) as a function of response mode, group, and emotion.

	Approach			Avoidance	
Group	Emotion	Mean RT	SD	Mean RT	SD
German	Happiness	766	(173)	821	(209)
	Fear	791	(192)	827	(217)
Turk/Middle-Eastern	Happiness	765	(190)	797	(201)
	Fear	757	(196)	795	(200)
Young	Happiness	702	(145)	735	(176)
	Fear	732	(149)	754	(183)
Old	Happiness	706	(176)	723	(139)
	Fear	703	(175)	697	(131)

### Conceptual Replication

**FIGURE 2:**
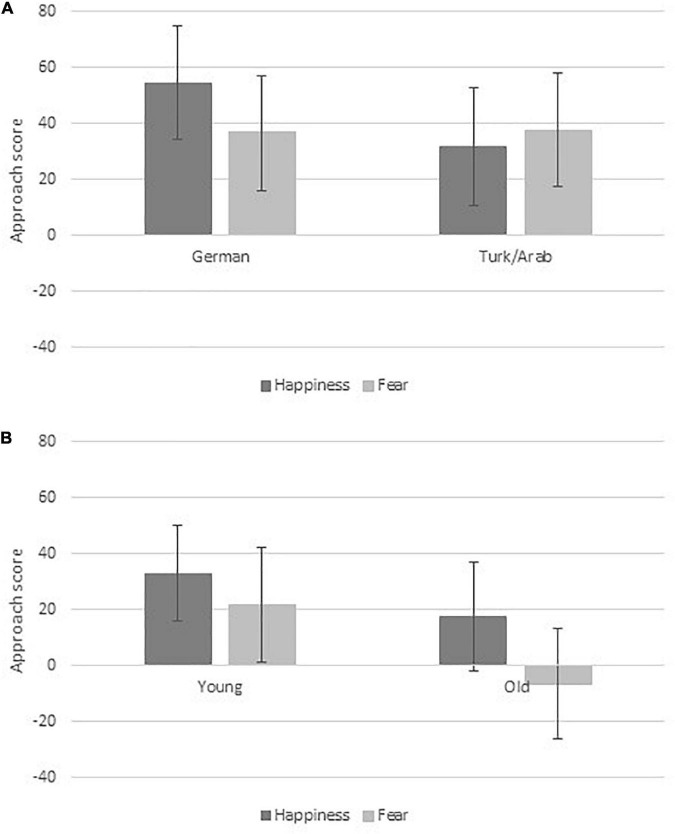
The approach scores (i.e., the difference in mean RT between approach and avoidance responses; whiskers are standard errors) for **(A)** the 2 (ethnicity: German vs. Turk/Midle-Eastern) × 2 (emotion) design and **(B)** the 2 (age: young vs. old) × 2 (emotion) design.

**Appendix Figure B1:**
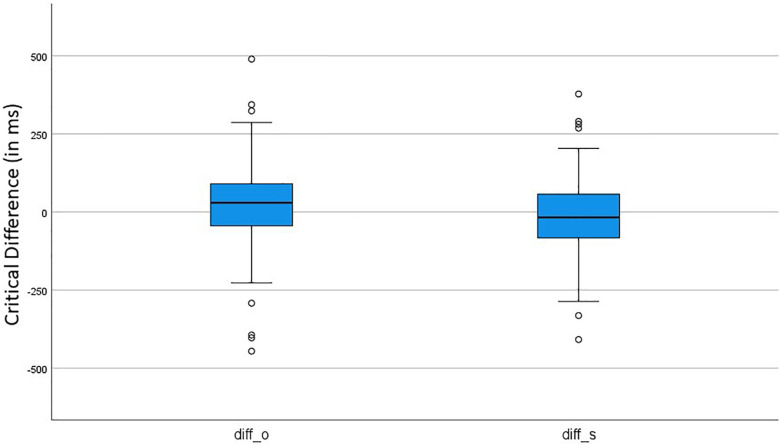
Boxplots for the critical differences. diff_o = approach scores for happy/German, fearful/Turk (averaged) minus approach scores fearful/German, happy/Turk (averaged); diff_s = approach scores for happy/young, fearful/old (averaged) minus approach scores fearful/young, happy/old (averaged).

First of all, we focused on the replication part of the study, that is, the 2 (ethnicity: German/Dutch vs. Turk/Middle-eastern) × 2 (emotion: happiness vs. fear) × 2 (approach vs. avoidance) design, as both theories – SMA and PCA – predict an interaction for this part of the design. As can be seen in Figure 2A, the pattern of means corresponds to the prediction of the two theoretical accounts: For Caucasian stimuli, the approach score for happy faces exceeds the one for fearful faces (Figure 2A, left); the pattern reverses for Turk/Middle-eastern faces (Figure 2A, right). With regard to inferential statistics, we followed the rationale outlined in our power planning (see Section “Participants”), that is, we build on the equivalence of the *F*-test (with *df_*N*_* = 1) for the interaction to a one-sample *t*-test to allow for one-tailed testing (Maxwell et al., 2017). The critical difference variable was: approach scores for happy/German, fearful/Turk (averaged) minus approach scores fearful/German, happy/Turk (averaged). This difference variable was burdened by outliers at both tails of the distribution (see Appendix Figure B1); thus, assumption of normality was violated (*p* = 0.003 according to a Shapiro–Wilks test). We therefore conducted a robust one-sample *t*-test (function *yuen.t.test* from the R package *PairedData*; Champely, 2018; see Wilcox, 2013, with regard to robust testing) with a trimming of γ = 0.2, which yielded *t*(37) = 2.17, *p* = 0.018 (one-tailed), *d_*Z*’_* = 0.30 (see Algina et al., 2005). The trimmed mean was *M*_t_ = –32 ms.^4^ The simple effects – the difference in approach scores for happiness and fear for the two groups – were not significant: *t*(37) = 0.95, *p* = 0.173 (one-tailed) for German/Duch and *t*(37) = –0.96, *p* = 0.171 (one-tailed) for Turk/Middle-eastern.

### Comparing Caucasian-Middle Eastern With Young–Old

As can be seen in Figure 2B, the pattern of means for the old-young comparison clearly deviates from the one for the German–Turk/Middle-eastern comparison. Again, the critical difference variable (i.e., approach scores difference happy–fearful for young minus approach scores difference happy-fearful for old) was burdened by outliers at both tails of the distribution (see Appendix Figure B1); assumption of normality is violated (*p* = 0.013 according to a Shapiro–Wilks test). A robust one-sample *t*-test (see above) yielded a trimmed mean of *M*_t_ = –18 ms, that is, the critical difference had the wrong sign (if we proceed from PCA). (A two-tailed exploratory test yielded, *t*(37) = –0.73, *p* = 0.466, *d*_*Z*_ = 0.09.)

The pattern of means numerically corresponds to two main effects (see Figure 2B): Average approach scores for happy faces were larger than those for fearful faces; this difference was indeed significant, *t*(61) = 2.13, *p* = 0.037, *d_*Z*_* = 0.27. Numerically, average approach scores for young faces were larger than those for old faces; this difference, however was not significant, | *t*| < 1.

To test for the higher order interaction (i.e., whether the interaction pattern for German–Turk/Middle-eastern significantly deviates from the pattern for old-young), we conducted again a robust paired samples *t*-test to compare the trimmed means of the two critical difference variables, that is, the one for Turk/Middle-eastern and the one for young/old, which yielded *t*(37) = 2.57, *p* = 0.007 (one-tailed), *d_*Z*’_* = 0.33 (*z* = 1.88, *p* = 0.030, one-tailed, for a Wilcoxon test; see Footnote 3).

## Discussion

For the other-relevant out-group/in-group comparison (i.e., Caucasian vs. Turk/Middle eastern young men), we replicated the result of Paulus and Wentura (2014); that is, for in-group expressers, happiness was associated with a higher approach score than fear; this pattern was reversed for out-group expressers. For the possessor-relevant out-group/in-group comparison (i.e., old persons vs. young persons), we found no moderation by group but a main effect for emotional expression (i.e., for both out-group and in-group expressers, happiness was associated with a higher approach score than fear). The overall interaction (i.e., the test whether the pattern was different for the two types of groups) was significant as well.

Both theories under debate – that is, the SMA as well as the PCA – can explain the pattern for the other-relevant out-group/in-group comparison. However, the prediction for the possessor-relevant out-group/in-group comparison diverges: The PCA would have predicted an interaction in this case as well since two valent features – that is, age and emotion – are present in each stimulus which either match or do not match. However, the possessor-relevant out-group/in-group comparison yielded no interaction of the two valent features. Instead, we found only the typical result of larger approach scores for the positive emotion compared to the negative emotion.

Where do we stand now with regard to the comparison of SMA and PCA? In Table 2, we present a synopsis of the hitherto found results. The results found with the approach-avoidance task by Paulus and Wentura (2014) were claimed as positive evidence by both accounts. The experiment described in the present article, however, shifts the balance in favor of SMA, as we have argued above.

**TABLE 2:** Evidence for the social message account (SMA) and the processing conflict account (PCA).

Paradigm	SMA	PCA
Approach–Avoidance Task	**Paulus and Wentura (2014)** **Wentura and Paulus, this article**	Paulus and Wentura (2014) *[Kozlik and Fischer, 2020]*
Evaluative priming	**Weisbuch and Ambady (2008)**	Craig et al. (2014) Paulus and Wentura (2018)
EAST	**Gurbuz et al. (2022)**	
Startle	Paulus et al. (2019)	
“Stroop”-variant	Kozlik and Fischer (2020, Experiments 1, 3, 4b) *[Wentura and Paulus, this article]*	**Kozlik and Fischer (2020, Experiments 1, 3, 4b)**
“Emotional Stroop”-variant	Kozlik and Fischer (2020, Experiments 2, 4a) *[Wentura and Paulus, this article]*	**Kozlik and Fischer (2020, Experiments 2, 4a)**

*Cell entries in bold are studies claimed as evidence by the respective authors for the theoretical account of the respective column. The remaining entries are either counter-evidence or reinterpretations (by the authors mentioned in italics) of the cited (bold) evidence. The black coloring is a weighting of evidence by the present authors in favor of the column theory (see text for further explanation).*

For the evaluative priming paradigm, we consider the initial results by Weisbuch and Ambady (2008) supporting the SMA as not replicable. This conclusion is based on the experiments of Craig et al. (2014) and Paulus and Wentura (2018). Interestingly, Kozlik and Fischer (2020) do not cite the recent evaluative priming results found by Craig et al. (2014) as well as Paulus and Wentura (2018), although they are more compatible with the PCA than with the SMA: They fit to the basic assumption of Kozlik and Fisher, namely the reasoning that two affectively connoted features of a face (i.e., emotion and group) are independently extracted and independently influence the target-related response. This is exactly what was found in the priming paradigm, in which the priming effect showed an effect of group membership and emotional expression, but no interaction. However, the results are not compatible with the second step of PCA, namely that the match or mismatch of the two features influences the following reactions (in this case, happy in-group faces, and fearful out-group faces should act as a positive prime and happy out-group faces and fearful in-group faces as a negative one). Of course, one could additionally assume that the specifics of the priming paradigm (e.g., very brief presentation of the primes, task irrelevance of the primes, direct correspondence of emotion and ethnic valence with the target-related response modes) preclude this step. However, this is only a speculative *post hoc* explanation which would need empirical validation. Nevertheless, the gray-shaded cell in Table 2 indicates that the evaluative priming evidence favors more the PCA over the SMA. Even if a moderator will be found in future research that predicts whether the SMA or the PCA pattern will be obtained in evaluative priming, we can clearly state that there is at least one context – the task as used by Craig et al. (2014) and by Paulus and Wentura (2018) – in which the two valent features are *not* immediately integrated to form a social message.

A potential hint to the cause of this failure to find a SMA-compatible pattern in the evaluative priming study is given by the EAST study of Gurbuz et al. (2022) that found such a pattern. Both paradigms aim to assess involuntarily activated valence associations. And in both the evaluative priming task as well as in the EAST, the two valent features are task-irrelevant. However, the facial expression itself is task-relevant in the EAST (since the side of the blurring has to categorized) but not in evaluative priming. This leads to the working hypothesis that stimuli presented in a situation that is completely devoid of a social character will not produce social messages. It is up to further research to test this hypothesis more thoroughly.

Until now, we did not discuss whether PCA can predict the EAST results as well. Unfortunately, Kozlik and Fischer (2020) did not have had the chance to discuss these findings because the experiment was conducted after publication of their article. Gurbuz et al. (2022) argued that there are three possible predictions for the EAST in light of the PCA. First, if one considers for a moment the evaluative decision trials (i.e., the word trials) of the EAST as irrelevant filler trials, the experiment by Gurbuz et al. (2022) was an almost one-to-one replication of Experiment 2 of Kozlik and Fischer: The faces varying in emotional expression and ethnicity were presented with right- versus left-blurring; task was to categorize the side of the blurring. Thus, proceeding from PCA, one would expect the same result as Kozlik and Fischer in their Experiment 2: Collapsed over “positive” and “negative” keys, responses in conflict trials should be slower than non-conflict trials; thus, an emotion × ethnicity interaction should have appeared. However, Gurbuz et al. (2022) did not find evidence for this. Second, if we put the EAST in line with the evaluative priming studies, Gurbuz et al. (2022) should have found an emotion × valence interaction and an ethnicity × valence interaction. This was not the case. Third, one could argue in principle that the match or mismatch of the two valent features determines the effective valence of the stimulus in the EAST. This would mean, however, that the combination of two negative features constitutes a positive stimulus. Only with this not very plausible assumption, the prediction of the PCA is the same as that of the SMA. Thus, we weight the EAST study as evidence for SMA.

The startle experiment by Paulus et al. (2019) was interpreted as evidence for SMA by the authors. Kozlik and Fischer (2020) did not discuss this article (maybe because the earlier article was published while the latter manuscript was already finished). Nevertheless, for two reasons we do not consider it as unequivocal evidence for SMA in the competition with PCA. First, Kozlik and Fischer (2020) can easily apply their argument that the “affective congruence status matters” to the results pattern found by Paulus et al. (2019): affective incongruent stimuli cause an amplification of the startle reaction. Second, from their viewpoint, Kozlik and Fischer (2020) would certainly address the fact that in the Startle experiment fear expressions and anger expressions produce almost exactly the same results (i.e., relative amplification of startle by in-group faces compared to out-group faces). Whereas Paulus et al. (2019) have to concede that auxiliary assumptions are needed for the angry faces, it is part of Kozlik and Fischer’s (2020) story to predict comparable results for fear and anger expressions. Thus, we refrain from categorizing the startle experiment as more “pro SMA” than “pro PCA” in Table 2.

We will now discuss at length the Stroop-like paradigm introduced by Kozlik and Fischer (2020). To recapitulate, Kozlik and Fischer expected and found an emotion × ethnicity interaction (with response times as dependent variable) in an experiment with the task to categorize the emotion as positive or negative. This result can straightforwardly be interpreted from the PCA perspective by the assumption that the task-irrelevant ethnicity feature is either congruent or incongruent to the target-related response. Moreover, the data include “signatures” of response interference paradigms, that is, a congruence sequence effect (Experiments 1 and 4b) and a congruence proportion effect (Experiment 3). As a contrast, SMA would predict that in-group happiness and in-group fear are unequivocally positive and negative because of their respective social messages. Hence, categorization of emotion should be easy. Out-group happiness and out-group fear are ambiguous with regard to the response: Nominally, happiness required a positive response although SMA predicts that out-group happiness is a negative stimulus; the mirror-image logic applies to fearful out-group expressions. Thus, in the paradigm by Kozlik and Fischer (2020), the SMA would have predicted a main effect of ethnicity (with slower responses toward out-group faces). In Experiment 1a, fear was used as the negative emotion. The main effect of ethnicity was not reported by Kozlik and Fischer (2020). However, the existence of an emotion × ethnicity interaction (which would be not predicted by SMA, but by PCA) indicates that PCA-predicted processes play a role. Interestingly, the picture changes if anger is used as the negative emotion, as was the case in Experiment 1b and Experiment 3. Kozlik and Fischer (2020) argue that for PCA, all negative expressions are exchangeable with regard to the hypotheses; for SMA, however, fear and anger make a difference: The social message of fearful in-group and out-group members differ (see above); this does not hold for anger. From a SMA perspective we can now apply the following logic: SMA does not predict differences in behavior for in-group and out-group anger, therefore, the prediction of a group main effect (which was hypothesized in the case of fear) in the Stroop-like paradigm changes to an interaction prediction (if anger is used). One might now argue that the exact form of the interaction pattern predicted by PCA (disordinal) and SMA (ordinal; only out-group joy will stand out in the 2 × 2 table) is different. However, this argument is not a strong one: a 2 × 2 interaction can adopt different patterns of means by adding or subtracting main effects (see, e.g., Wentura and Degner, 2010b). However, even in this case, SMA has neither a good argument in favor of the finding of a congruence sequence effect nor in favor of finding the same pattern with averted faces (Experiment 4b).^5^ In sum, we would acknowledge that the Stroop-like results favorize PCA over SMA. If this interpretation holds, a second context – the first one was the evaluative priming context (see above) – is identified that does not lead to social message effects. Of course, our working hypothesis given above about the failure to find social message effects in evaluative priming cannot be applied here since the facial stimulus is as task-relevant here as it is in the EAST, the approach-avoidance task, and the startle paradigm. What differs is the fact that the emotional expression is the task-relevant feature (which is not the case in any of the other experiments). Thus, a second working hypothesis can be formulated stating that targeting the emotional expression itself leads to a separation of the two features (or in other words: it hinders an integration). The reason for this might be the process behind emotion identification: Emotional expressions have to be categorized on the basis of specific muscle configurations; these are not different for different ethnicities. However, for fast categorization attentional focus might be on these features. If incidentally the ethnicity feature is processed, it is either in contrast or in accordance with the response that has to be given.

Finally, we should discuss the “Emotional Stroop” variant introduced by Kozlik and Fischer (2020). In contrast to the authors, we see the balance here more in favor of SMA than PCA for three reasons. If the effect found in Experiment 2 turns out to be a robust one, SMA has an explanation that is at least as plausible than the one derived to PCA. However, our view on the EAST paradigm from the PCA viewpoint (i.e., that the EAST experiment by Gurbuz et al., 2022, includes a failed replication of Experiment 2 by Kozlik and Fischer, 2020) already gives a hint to a replication problem. Moreover, Experiment 4a – the experiment with averted faces – does not show any evidence for the emotion × ethnicity interaction. This perfectly fits SMA but is in contrast to PCA.

## Conclusion

We started by the simple question whether two evaluative features of faces – particularly emotional expression and ethnicity – are initially processed independently (and may interact at later stages of processing to influence behavior) or whether they are immediately integrated to form a social message based on both pieces of information. As it is often the case with such binary questions: It depends! At this point in time, we would summarize the evidence as: There are results that can be explained by both the SMA as well as the PCA; there are results that can easily be explained by PCA, but not SMA, and, finally, there are results that fit SMA, but not PCA. It seems as if the task for future research is not which account comes true but to identify moderator variables that determine which process dominates.

## Data Availability Statement

The datasets presented in this study can be found in online repositories. The names of the repository/repositories and accession number(s) can be found below: https://osf.io/hp7rd/.

## Ethics Statement

The studies involving human participants were reviewed and approved by Ethics Committee of the Faculty of Human and Business Sciences (Saarland University, Germany). The patients/participants provided their written informed consent to participate in this study.

## Author Contributions

AP and DW designed the experiment, analyzed the data, and revised the manuscript. AP programed the experiment and collected data. DW wrote the draft manuscript. Both authors contributed to the article and approved the submitted version.

## Conflict of Interest

The authors declare that the research was conducted in the absence of any commercial or financial relationships that could be construed as a potential conflict of interest.

## Publisher’s Note

All claims expressed in this article are solely those of the authors and do not necessarily represent those of their affiliated organizations, or those of the publisher, the editors and the reviewers. Any product that may be evaluated in this article, or claim that may be made by its manufacturer, is not guaranteed or endorsed by the publisher.

## Footnotes

1 The index “a” and “b” is not used by Kozlik and Fischer (2020). In their report, Experiment 4 consisted of two blocks of trials with averted faces. In the first block (“4a” in our notation), direction of faces is task-relevant; therefore it is a conceptual replication of Experiment 2, now with averted faces. In the second block (“4b” in our notation), emotion of faces is task-relevant; therefore it is a conceptual replication of Experiment 1 with averted faces. Since sequence of blocks was not counter-balanced, at least Experiment 4a can be interpreted as if it had been an isolated experiment. From the viewpoint of Kozlik and Fischer (2020) the following higher order interaction in Experiment 4 was interpreted as evidence for PCA: The replication of Experiment 1 (i.e., Experiment 4b) shows – as predicted – a congruence sequence effect (see above), which is – again, as predicted – missing in the replication of Experiment 2 (i.e., Experiment 4a). However, the authors have to admit that this pattern is constrained to right-looking faces.

2 As most of the studies cited in the Introduction, we refrained from varying the group status of the participants (i.e., we did not additionally recruit Turkish/Middle-eastern young men or old people). For both PCA and SMA it is essential that the outgroup is a negatively connoted out-group (e.g., an ethnicity that is met with prejudice). Therefore, asymmetric reaction patterns may occur when two different social groups are presented with in-group vs. out-group faces (see, e.g., Zhang et al., 2021).

3 ^3^If we assume the standard deviations of the critical differences of the two blocks to be the same, we can argue that the difference variable in the other-relevant negativity block has a (standardized) mean of *M* = *d*_Z(other–relevant block)_ with SD = 1 whereas the corresponding difference variable for the possessor-relevant negativity block has a mean of *M* = 0 with SD = 1. Therefore the higher order difference variable has a mean of *M* = *d*_Z(other–relevant block)_ –0. The variance of the higher order difference variable is the sum of the two variances of minuend and subtrahend if – second assumption – the correlation between minuend and subtrahend is zero (which is plausible in the present context); hence the standard deviation is square-root(2) and the effect size of the interaction is *d*_Z(interaction)_ = *d*_Z(other–relevant block)_/square-root(2).

4 An alternative approach to account for non-normality would have been to compare the two averages in a Wilcoxon test (e.g., Blair and Higgins, 1985); it was significant as well, *z* = 1.90, *p* = 0.029 (one-tailed).

5 Experiment 4b, however, provide no strong evidence at all because the PCA-predicted results were only found in an exploratory *post hoc* analysis that took the face direction (right/left) into account. The predicted pattern was found for right-looking faces only.

## Appendix A

**Appendix Table A1:** Categorization and ratings of the four stimulus sets (standard deviations in parentheses).

	German		Turkish		Young		Old	
Emo. Categ. (% correct)								
Angst	72	(2.06)	68	(1.95)	72	(2.28)	66	(2.38)
Freude	93	(0.16)	93	(1.14)	98	(0.48)	97	(0.97)
Intensity								
Angst	5.32	(0.35)	5.33	(0.49)	5.38	(0.77)	5.38	(0.12)
Freude	4.78	(0.27)	4.69	(0.45)	5.07	(0.49)	4.85	(0.53)
Unambigueness								
Angst	5.20	(0.53)	4.79	(0.74)	4.92	(0.53)	4.85	(0.64)
Freude	6.07	(0.27)	5.77	(0.32)	6.02	(0.42)	5.86	(0.34)
Naturalness								
Angst	4.28	(0.54)	4.39	(0.40)	4.01	(0.46)	4.58	(0.42)
Freude	5.13	(0.56)	5.21	(0.70)	5.31	(0.98)	5.57	(0.40)
Dominance								
Angst	3.66	(0.34)	4.10	(0.67)	3.61	(0.23)	4.25	(0.79)
Freude	3.94	(0.51)	4.04	(0.60)	3.55	(0.39)	4.18	(0.40)
Sociability								
Angst	3.72	(0.40)	4.21	(0.29)	4.07	(0.48)	3.56	(0.47)
Freude	4.81	(0.33)	5.15	(0.41)	5.31	(0.56)	5.20	(0.60)
Typicality	1.82	(0.49)	6.14	(0.46)	2.07	(0.80)	1.59	(0.28)
Estimated age	28	(3.42)	28	(3.85)	27	(2.78)	70	(2.44)
Attractiveness	3.01	(0.81)	3.28	(0.63)	4.34	(0.87)	3.01	(0.77)

*Emotion categorization: response categories: joy, disgust, fear, sadness, surprise, contempt, anger, neutral, other; intensity: 1-‘very weak’ to 7-‘very intense’; unambigueness: 1-‘very ambiguous’ to 7-‘very unambiguous’; naturalness: 1-‘very posed’ to 7-‘very natural’; dominance: 1-‘very submissive’ to 7-‘very dominant’; sociability: 1-‘very shy’ to 7-‘very sociable’; typicality: 1-‘typically German’ to 7-‘typically Arabic’; attractiveness: 1-‘very unattractive’ to 7-‘very attractive’.*
